# Magnetic Resonance Imaging-Guided Radiation Therapy for Early-Stage Gastric Mucosa-Associated Lymphoid Tissue Lymphoma

**DOI:** 10.7759/cureus.29035

**Published:** 2022-09-11

**Authors:** Neris Dincer, Gamze Ugurluer, Gorkem Gungor, Teuta Zoto Mustafayev, Banu Atalar, Enis Ozyar

**Affiliations:** 1 Radiation Oncology, Acibadem Mehmet Ali Aydinlar University School of Medicine, Istanbul, TUR; 2 Radiation Oncology, Acibadem Maslak Hospital, Istanbul, TUR; 3 Radiation Oncology, Acibadem Hospital, Istanbul, TUR

**Keywords:** mucosa-associated lymphoid tissue, radiotherapy, intensity modulated radiotherapy, deep inspirium breath hold, dibh, imrt, adaptive radiation therapy, mr-linac, maltoma

## Abstract

Lymphoid neoplasia derived from mucosa-associated lymphoid tissue (MALT; also abbreviated as MALToma) is most commonly seen in the stomach. Radiotherapy (RT) is indicated in early-stage disease as a standard of care. With the advent of RT techniques, large field irradiation was replaced by involved site and involved field approaches. Magnetic resonance imaging-guided online adaptive RT (MRgRT) has the advantage of better soft tissue visualization, adaptive planning before each fraction, and online tumor tracking during treatment; hence, it could be a safe and effective choice for gastric MALToma patients. Herein, we investigated the interfractional changes in target and the impact of MRgRT on daily dosimetry in a gastric MALToma case. A patient diagnosed with MALToma who failed to respond to antibacterial treatment was referred to our clinic for RT. He was found to be suitable for MRgRT. We treated the patient with MRgRT in 20 fractions to a total dose of 30 Gy. Reoptimized adaptive plans were generated before each fraction since the coverages of the original plan were inadequate in each fraction. The patient showed good compliance and tolerated the treatment well. To our knowledge, this is the first documented case of a gastric MALToma treated with MRgRT. MRgRT is safe and feasible for this patient group with improved target coverage using small planning target volume margins. Without online adaptive planning, the target coverages would be inadequate and we would risk surrounding tissues to get higher doses.

## Introduction

The neoplastic proliferation of B cells in the mucosa-associated lymphoid tissue (MALT) is referred to as MALT lymphoma, which is also denoted as MALToma. The most frequently involved organ is the stomach [1]. Gastric MALT lymphoma (GML) constitutes 38% of primary gastric lymphomas [2], and it has a well-established relationship with *Helicobacter pylori* (HP) infection [3]. After the discovery of this relation, HP eradication became the standard of treatment for GML [4] and there are groups advocating that it should be given irrespective of the HP status and the stage of the disease [5]. Surgery, radiotherapy (RT), and chemotherapy remain other options for unresponsive, recurrent, or advanced stage GML [6].

RT is indicated in stage I-II patients who fail to respond to HP eradication treatment [7]. RT was found to be associated with excellent local control rates as the primary treatment or after HP eradication [8,9]. However, the daily change of stomach size, shape, motion as well as surrounding organs at risk remains challenging [10].

Herein, we report a case of GML that we treated with magnetic resonance imaging-guided online adaptive radiotherapy (MRgRT). To our knowledge, this is the first report documenting the MRgRT treatment of a GML case.

## Case presentation

The patient is a 76-year-old male. He has coronary artery disease, cardiac arrhythmia, hypertension, and urolithiasis. He underwent surgeries for prostate hyperplasia and nephrolithiasis 10 years ago and he had a coronary angiography with stent placement. He takes oral anticoagulants, antihypertensive, antihyperlipidemic, and anxiolytics. The patient presented to a gastroenterologist with the complaint of a burning sensation in the stomach ongoing for the past two years. History revealed that the patient had gastroscopy and colonoscopies done routinely and the biopsy results of the last gastroscopy that was done one year ago revealed no proof of malignancy. An abdominal computed tomography (CT) report revealed no pathological findings except for dilatation in intrahepatic bile ducts and a hypervascular lesion in the liver. Gastroscopy was performed to investigate the etiology of the patient's complaints. Macroscopic findings were consistent with erosive gastritis and granular-type polyps in the fundus. Samples were taken and sent to the pathology department. The morphological evaluation showed diffuse lymphoid infiltration and antigenic evaluation was consistent with a negative cluster of differentiation (CD)3, CD10, and CD23, positive CD20, and weak-positive CD5. Kappa-light chain was found to be focally positive. Antigenic and morphological features were found to be consistent with a low-grade MALT lymphoma with CD5 positivity. HP immunohistochemistry was negative. The patient was then sent to positron emission tomography and computed tomography (PET-CT) scan for staging. PET-CT showed the primary lesion located at the greater curvature with a dimension of 4 cm and a maximum standard uptake value (SUVmax) of 6.2. The endoscopic ultrasonography (EUS) revealed no pathological lymphadenopathy with a EUS stage of IE. The work-ups affirmed that the patient had stage IE gastric MALT lymphoma. The patient was started on antibiotic treatment.

The control gastroscopy showed that the lesion did not regress during the last two months. Antibiotic treatment was deemed to fail so the patient presented to our clinic for treatment. The patient was discussed in our multidisciplinary tumor board and the board decided to proceed with RT. We decided to treat the patient with MRgRT.

Simulation CT, PET-CT, and planning TRUFI three-dimensional 0.35 Tesla magnetic resonance imaging (MRI) through MRIdian® Linac (ViewRay Inc., Mountain View, CA) were acquired. A deep inspiration breath hold (DIBH) MRI image was acquired in 17 seconds without a contrast agent. MRI scan was fused with PET-CT and gross tumor volume (GTV) was delineated as stomach and proximal duodenum. A margin of 7 mm was given to GTV to create a planning target volume (PTV). Organs at risk (OARs) were determined as the right kidney, left kidney, spinal cord, liver, large bowel, aorta, inferior vena cava, pancreas, esophagus, and duodenum. Step-and-shoot intensity-modulated radiotherapy (IMRT) treatment plan was generated. Departmental dose constraints were applied. The reference plan had 24 fields and 93 segments with 6 MV flattening filter-free (FFF) photons in a single isocenter. A total dose of 30 Gy in 20 fractions (1.5 Gy/fraction) was prescribed for the target volume. The patient was instructed not to eat or drink starting three hours prior to simulation and treatment to prevent significant interfractional stomach variations.

Furthermore, a three-dimensional (3D) high-resolution MRI view was obtained in each fraction and the anatomy of the day was evaluated by a radiation oncologist. GTV and OARs were contoured accordingly. An online adaptive plan was generated and re-optimization was performed in all fractions due to mainly lack of target volume coverage.

**Figure 1:**
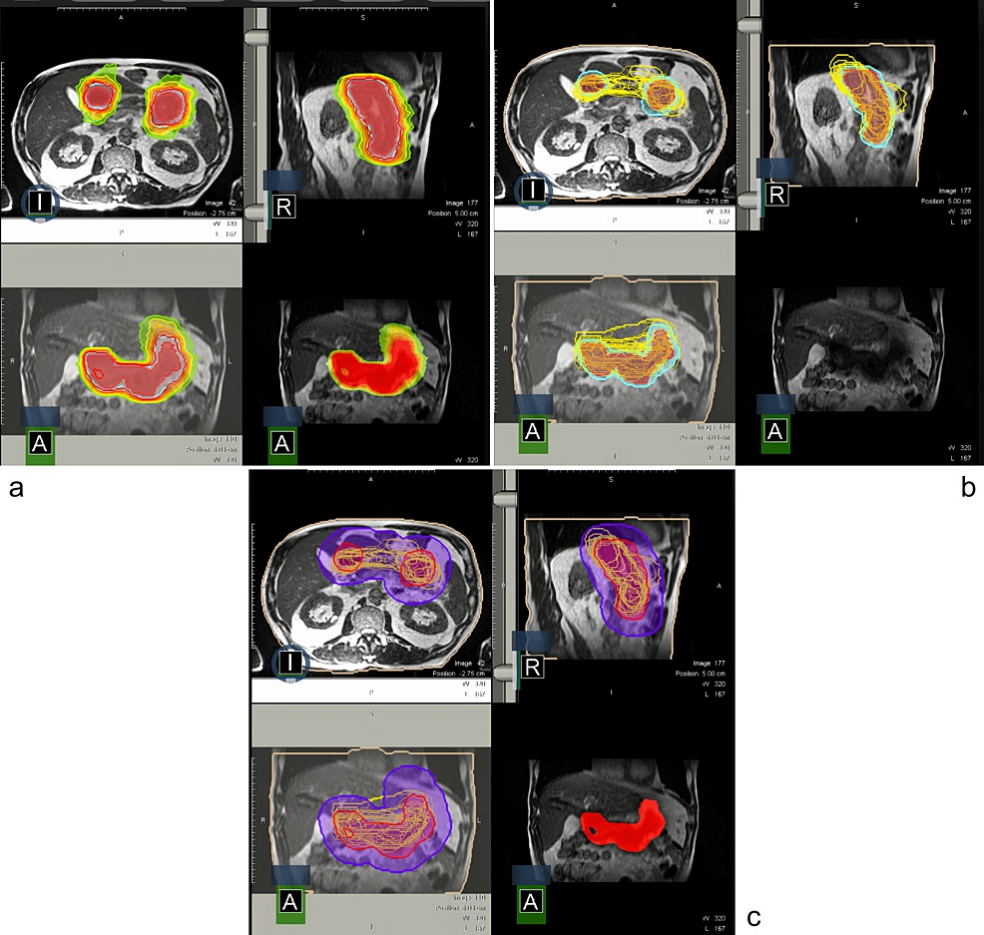
Representation of contours and the change over the fractions A: Isodose color-wash illustration of GTV. The red area is 100% and the yellow area is 50% isodose line. B: The change of GTV over the fractions. The red area is the GTV of the original plan and the blue line represents the original PTV. Yellow lines demonstrate the change of GTV in between fractions. C: The purple area represents a fused version of all GTV contours over the treatments. We deduce that a PTV of 3 cm margin would cover the change in the daily anatomy if an adaptive plan was not generated. GTV: gross tumor volume; PTV: planning target volume.

**Figure 2:**
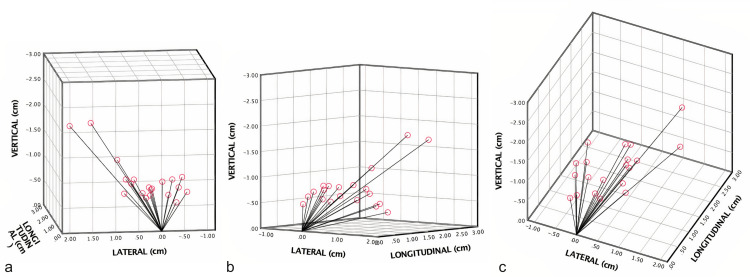
Three-dimensional representation of vectoral change in GTV coordinates over the course of fractions The change in the vectoral coordinates of GTV contours from vertical, lateral, and longitudinal views are shown in images A, B, and C, respectively. GTV: gross tumor volume; cm: centimeter.

**Figure 3:**
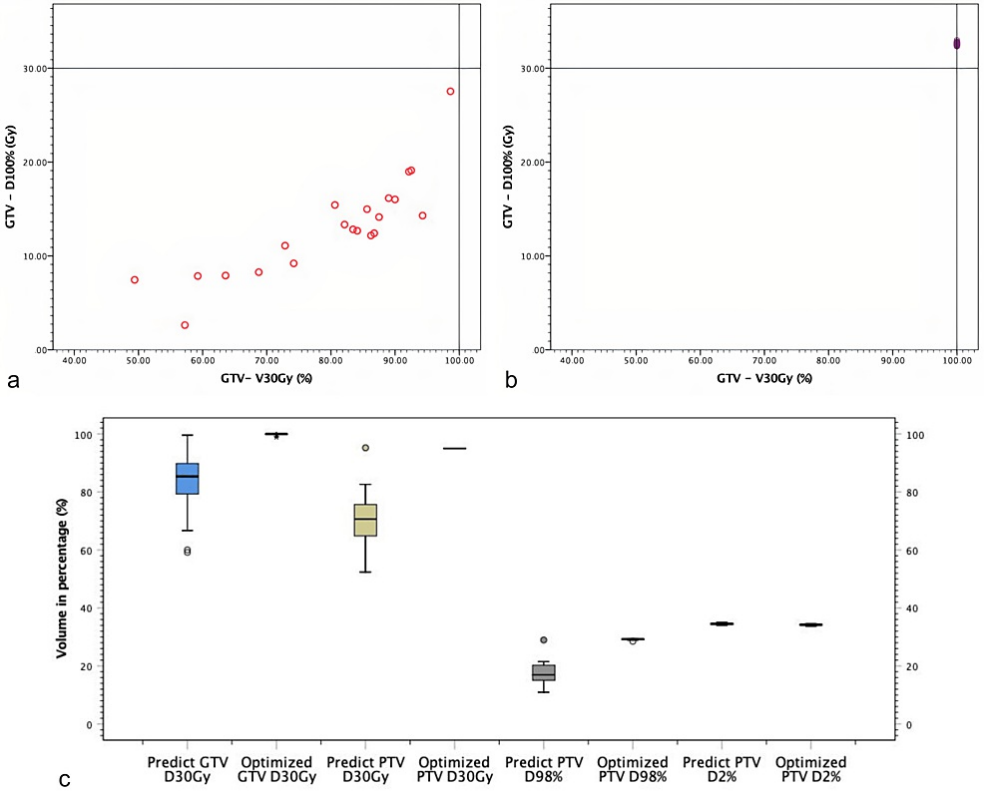
Graphical illustration of predicted and reoptimized plan GTV coverage A: Change of GTV volume percentage receiving 30 Gy over the course of fractions if the original plan was applied for each fraction. B: GTV volume percentage receiving 30 Gy when the reoptimized plan was applied for each fraction. C: Comparison of D30Gy of GTV, PTV, D98% of PTV, and D2% of PTV in predicted plans and reoptimized plans. GTV: gross tumor volume; PTV: planning target volume; Gy: gray; Dmax: maximum dose; Dmean: mean dose; Dmin: minimum dose; V100%: percentage volume receiving 100% of prescribed dose; D98%: minimum dose covering 98% of the target volume; D2: minimum dose covering 2% of the target volume.

Figure 1 shows the GTV and PTV planning dose coverage in color-wash, inter-fractional displacement shifts of GTV during treatments, and required PTV margin to cover GTV for all fractions if adaptation was not performed, respectively. If the patient underwent each fraction according to the predicted plan, GTV coverage would be inadequate, and we would have to give a safety margin of 3 cm for the PTV. Moreover, Figure 2 demonstrates the 3D center of the mass shift of GTV per fraction. Figure 3 shows the dose coverages of predicted and adaptive plans.

The target volume was tracked in a single sagittal plane with four frames per second by using a 3 mm boundary margin for DIBH auto beam on and off gating.

The patient tolerated the treatment well. He did not have any grade 3+ toxicity but mild nausea for which we prescribed ondansetron. The patient is now well and comes to his regular follow-up visits.

## Discussion

Extranodal lymphomas constitute 40% of non-Hodgkin's lymphomas (NHL) and the most common origin is the stomach [11,12]. The two most common types of gastric lymphomas are diffuse large B-cell lymphoma (DLBCL) and marginal zone B-cell NHL of the MALT type [13]. GMLs are low-grade lesions bearing a well-established relationship with HP infection [14]. Given this knowledge, it is recommended that all early-stage GML patients start on HP eradication treatment irrespective of their HP status since some HP-negative patients are reported to benefit from the regimen [5].

RT is indicated in patients who fail to respond to HP eradication treatment [15] or it can be the first-line treatment in HP-negative patients [16]. It can also be given after the HP eradication treatment without waiting for treatment response [16]. Current European Society for Medical Oncology (ESMO) guidelines for marginal zone lymphomas advise that patients who fail to respond to HP eradication, those with overt progression, deep invasion, and t(11;18) translocation undergo RT [17]. Earlier on, the whole abdomen was irradiated with an optional boost to involved fields [18]. With time, it was understood that GML tended to be localized so this “extended-field” approach was abandoned [19] and replaced by involved-field radiotherapy (IFRT) in which the stomach and the first part of the duodenum are irradiated and perigastric lymph nodes are included only if involved [20].

The advances of new techniques led RT to achieve better results with a better conservation of the OARs. A comparative study analyzing 3D conformal RT (3D-CRT), IMRT, and volumetric modulated arc therapy (VMAT) for gastric MALT lymphoma found IMRT to be superior to the other modalities in terms of homogeneity index. IMRT and VMAT yielded better conformity index and lower dose to the liver than 3D-CRT. The authors deduced that IMRT yields the best plan quality for gastric lymphoma [21]. Another dosimetric study comparing 3D-CRT, step-and-shoot IMRT (SIMRT), VMAT, and tomotherapy plans for gastric MALT lymphoma patients deduced that IMRT plans resulted in lower doses to OARs, and DIBH (VMAT-DIBH and SIMRT-DIBH) technique was associated with smaller irradiated volume [22]. DIBH-IMRT was also shown to be associated with cardiac sparing [23].

Despite developed techniques, RT to the stomach remains a challenge due to OARs (namely, kidneys, liver, and small bowel) and the daily variations of the stomach’s position, size, and shape [10]. As aforementioned, the DIBH technique might be useful to ensure that the stomach is not affected by respiratory motion during treatment. Daily CT guidance was shown to improve target coverage in gastric MALT lymphoma RT. The authors also reported that gastric volume substantially varied between fractions despite DIBH and restricted oral intake [10]. However, CT images do not yield adequate visualization of the soft tissues, so anatomical landmarks other than the stomach, generally bony landmarks, are used for tumor tracking.

MRI-Linac is an MRI-based linear accelerator in which MRI images are taken and tumor tracking is performed with live image acquisition and MRgRT is performed with this device. MRgRT was found to be effective in liver and pancreas tumors in previous studies [24,25]. Superior target coverage with a higher biologically effective dose is achieved with MRgRT. Treatment of a gastric DLBCL with MRgRT to a dose of 30 Gy in 20 fractions was reported and the authors claimed that interfractional stomach variations reached up to 5.0 cm [26], showing the importance of precise target tracking.

An earlier study presented as a poster reported results of seven GML patients treated with image-guided radiotherapy (IGRT) with daily MRI acquisition. In 35% of cases, they detected a daily target shift greater than the PTV margin, and daily adjustments were performed accordingly. MRI images enable the detection of this variation as well as the formation of smaller PTV margins. However, in this study, the online adaptive approach was not used during treatment [27].

Herein, to our knowledge, we present the first documented case of gastric MALT lymphoma treated with MRgRT. MRgRT allowed us to be oriented with daily size, shape, and motion variations of the stomach as well as the motion and localization of surrounding OARs. We noticed that the reference plan may miss PTV coverage due to anatomical changes, but we performed daily adaptive planning, so we did not compromise on our PTV coverage. No dose violation was present during any of the fractions. The patient responded well to the treatment with no > grade 3 side effects. The patient showed good performance and has returned to his daily routine.

## Conclusions

RT has a foremost role in the treatment of gastric MALToma. Given the close proximity to intra-abdominal structures as well as the variabilities of the daily anatomy, irradiation of this field without compromising from target coverage requires daily set-up and/or motion control in which conventional CT-based imaging has some weaknesses. MRgRT has the advantage of target and healthy organ distinction and the device has a tumor tracking system that breaks irradiation if the target moves with inspiration, patient position, and changes in instant anatomy with organ motions. The plan adaptation based on daily anatomy leads to smaller PTV margins, which yield a better safety profile. Herein, we present the first reported case of a gastric MALToma treated with MRgRT and it can be safely used for this tumor.
